# Monitoring smart infusion pumps in adult intensive care units

**DOI:** 10.15649/cuidarte.5160

**Published:** 2026-04-21

**Authors:** Mario Andrés Narváez-Martínez, Andrea Rosas-Santana, Yudy Andrea Rojas-Castañeda, Diana Catalina Zapata-Cristancho

**Affiliations:** 1 Fundación Cardioinfantil-Instituto de Cardiología, Bogotá, Colombia. E-mail: manarvaezm@lacardio.org Fundación Cardioinfantil-Instituto de Cardiología Bogotá Colombia manarvaezm@lacardio.org; 2 Fundación Cardioinfantil-Instituto de Cardiología, Bogotá, Colombia. E-mail: arosas@lacardio.org Fundación Cardioinfantil-Instituto de Cardiología Bogotá Colombia arosas@lacardio.org; 3 Fundación Cardioinfantil-Instituto de Cardiología, Bogotá, Colombia. E-mail: jrojasc@lacardio.org Fundación Cardioinfantil-Instituto de Cardiología Bogotá Colombia jrojasc@lacardio.org; 4 Fundación Cardioinfantil-Instituto de Cardiología, Bogotá, Colombia. E-mail: dzapata@lacardio.org Fundación Cardioinfantil-Instituto de Cardiología Bogotá Colombia dzapata@lacardio.org

**Keywords:** Medication Therapy Management, Infusión Pumps, Patient Safety, Health Information Interoperability, Critical Care Nursing, Administración del Tratamiento Farmacológico, Bombas de Infusión, Seguridad del Paciente, Interoperabilidad de la Información en Salud, Enfermería de Cuidados Críticos, Administração de Tratamento Farmacológico, Bombas de Infusão, Segurança do Paciente, Interoperabilidade da Informação em Saúde, Enfermagem de Cuidados Críticos

## Abstract

**Introduction:**

Medication administration in intensive care units is one of the main care activities of nursing professionals. The integration of scientific knowledge, together with the use of technologies such as infusion pumps, enables the reduction of errors and improves the quality of care.

**Objective:**

To describe the use of an infusion pump monitoring platform for medication administration in adult intensive care units at a university hospital in Bogotá, Colombia.

**Materials and Methods:**

This was an observational, retrospective study that collected 35,738 medication administration records from five intensive care units using a monitoring platform during 2023. A descriptive analysis was performed using frequencies and proportions, along with simple associations assessing compliance between medications and doses.

**Results:**

Compliance with the drug library during the first year of the platform was 66%. The platform reported 5,589 hard-limit blocks involving high-alert medications, of which 76% were related to the administration of noradrenaline, midazolam, and fentanyl.

**Discussion:**

The results were consistent with other studies, demonstrating drug library compliance ranging from 65% to 80% during the first year of implementation.

**Conclusion:**

The implementation of institutional medication administration monitoring programs, using technologies integrated into infusion pumps, positively impacts patient safety, reduces costs associated with care, and continuous improvement within institutions.

## Resumen

**Introducción:**

La administración de medicamentos en unidades de cuidado intensivo constituye una de las principales actividades del cuidado en los profesionales de enfermería. La integración de conocimientos científicos, sumado al uso de tecnologías como bombas de infusión, permiten reducir errores y mejorar la calidad de la atención.

**Objetivo:**

Describir el uso de una plataforma de seguimiento de bombas de infusión en la administración de medicamentos en unidades de cuidado intensivo adulto de un Hospital Universitario de Bogotá, Colombia.

**Materiales y métodos:**

Estudio observacional, retrospectivo, el cual recolectó 35.738 registros de administración de medicamentos en cinco unidades de cuidado intensivo a través del uso de una plataforma de seguimiento durante el año 2023. Se realizó un análisis descriptivo utilizando frecuencias y proporciones, además de asociaciones simples de cumplimiento entre medicamentos versus dosis.

**Resultados:**

El cumplimiento a la biblioteca de medicamentos durante el primer año de la plataforma fue del 66%. La plataforma informó de 5.589 retrocesos en medicamentos de alto riesgo, de los cuales el 76% se relacionó con la administración de noradrenalina, midazolam y fentanilo.

**Discusión:**

Los resultados fueron consistentes con otros estudios, demostrando un cumplimiento a la biblioteca de medicamentos entre el 65% y 80% en su primer año de implementación.

**Conclusiones:**

La implementación de programas institucionales de seguimiento a la administración de medicamentos, mediante el uso de tecnologías integradas a las bombas de infusión, permiten impactar positivamente la seguridad del paciente, reducir costos asociados a la atención y fortalecer la mejora continua de las instituciones.

## Resumo

**Introdução:**

A administração de medicamentos em unidades de terapia intensiva é uma das principais atividades assistenciais dos profissionais de enfermagem. A integração do conhecimento científico, aliada ao uso de tecnologias como bombas de infusão, permite reduzir erros e melhorar a qualidade do cuidado.

**Objetivo:**

Descrever o uso de uma plataforma de monitoramento de bombas de infusão na administração de medicamentos em unidades de terapia intensiva para adultos de um Hospital Universitário em Bogotá, Colômbia.

**Materiais e métodos:**

Este foi um estudo retrospectivo e observacional que coletou 35.738 registros de administração de medicamentos em cinco unidades de terapia intensiva por meio do uso de uma plataforma de monitoramento durante o ano de 2023. Foi realizada uma análise descritiva utilizando frequências e proporções, bem como associações simples de adesão entre medicamentos e doses.

**Resultados:**

A adesão à biblioteca de medicamentos durante o primeiro ano de uso da plataforma foi de 66%. A plataforma relatou 5.589 recalls de medicamentos de alto risco, 76% dos quais relacionados à administração de norepinefrina, midazolam e fentanil.

**Discussão:**

Os resultados foram consistentes com outros estudos, demonstrando adesão da biblioteca de medicamentos entre 65% e 80% no primeiro ano de implementação.

**Conclusões:**

A implementação de programas institucionais de monitoramento da administração de medicamentos, utilizando tecnologias integradas a bombas de infusão, pode impactar positivamente a segurança do paciente, reduzir os custos associados ao cuidado e fortalecer a melhoria contínua nas instituições.

## Introduction

Before the 1970s, medications were administered via gravity infusion, a method in which the drip rate depended on gravitational force and was manually regulated^1^. Subsequently, conventional infusion pumps were developed that allowed infusion rates to be programmed only in ml/h and were intended for use in the administration of parenteral nutrition and cardiovascular medications^1^,^2^.

At the end of the 20th century, the Dose Error Reduction System (DERS) was integrated into infusion pumps. This integration was considered by the Emergency Care Research Institute (ECRI) in 2002 a requirement for the acceptance and recommendation of smart infusion pumps in intravenous medication administration therapy^3^. With this technological advance, infusion pumps could now support end users during medication infusion programming through the customization of drug libraries^2^. The advantages of this integrated software, within the framework of the National Patient Safety Goals proposed by the Joint Commission, include enabling the standardization of parameters related to concentration, safe dosing ranges, doses per unit of time, and duration for continuous and intermittent infusions of medications strategically prioritized by the medical center, as well as incorporating an alert system that notifies end users when programming falls outside the established limits^2^,^4^–^6^.

Smart infusion pumps are considered biomedical devices that integrate embedded software capable of hosting drug libraries, providing dose calculation support, and generating alerts for incorrect prescriptions, calculation errors, and programming errors^1^. These pumps enable systematic and objective data capture, thereby supporting the safe administration of medications. However, implementing smart pumps has been considered an arduous and complex process^2^,^4^.

Smart pump technology benefits patient safety and the healthcare professionals involved^6^. These advanced infusion devices create a controlled environment for the safe administration of medications through the appropriate use of drug libraries and the standardization of concentrations. By establishing precise programming of infusion rate and dose within safe limits, the risk of calculation errors made by staff and the incidence of adverse events among patients in clinical care settings, such as intensive care units, surgical services, and pediatrics, are minimized^2^,^5^,^7^. Among other safety-related benefits, smart infusion pumps allow the integration of multiple interoperable technological systems, which in turn enable data analysis aimed at identifying areas for improvement, accurate recording of medication infusion data in the medical record, automatic programming of infusions from the electronic health record, and coordination of infusion therapy through the use of barcodes^5^,^8^.

Adopting interoperable smart infusion pumps with monitoring software encounters technical and organizational barriers, such as outdated drug libraries due to delayed updates, ineffective alerts, usability issues, alarm fatigue, insufficient training, and a lack of clear processes for drug library management. These limitations, described across different hospital settings, are associated with low DERS compliance, equipment variability, and preventable safety risks. In contrast, interoperability and data analysis have been shown to reduce errors and improve efficiency, although they require context-specific implementation strategies. The absence or incomplete implementation of these technologies perpetuates medication errors, limits the prevention of adverse events, reduces efficiency, and increases healthcare costs^3^,^9^–^11^.

A limiting factor in the implementation of smart infusion pumps has been low drug library compliance. In addition, a recurring pattern of alert overrides has been observed, reflecting low user acceptance of these devices and highlighting the imperative need to integrate smart pumps with other technological systems^10^,^11^. This integration or interoperability between technological systems and infusion pumps is known as a “closed loop” medication management system and has been considered the latest innovation in safe medication administration. It ensures the interception of errors not only during programming, but also throughout medication administration, prescription, and transcription^2^,^5^,^10^,^12^,^13^.

Smart infusion pumps configured independently, despite incorporating DERS, may be affected by errors related to incorrect programming (based on preset dosing ranges), incorrect doses (that do not match the prescription order), incorrect medications, wrong patient selection, or incorrect administration instructions. In addition, smart pumps cannot detect changes in the patient's clinical condition, anticipated transfer orders, or unintentional changes in the concentration of a medication solution^8^,^14^,^15^.

Considering the above, it is evident that the implementation of smart infusion pumps involves adapting hospital infrastructure, supervising their use, ensuring adequate time availability, securing the engagement of multidisciplinary staff responsible for drug library standardization, and promoting effective communication between teams, in addition to ongoing operational and labor costs, which together may limit their benefits across different clinical units^3^,^9^.

The objective of this study was to describe the use of an infusion pump monitoring platform for medication administration in adult intensive care units (ICUs) at a university hospital in Bogotá, Colombia

## Materials and Methods

This was an observational, retrospective study with an analytical approach. The study population consisted of medication administration records collected through a smart infusion pump monitoring platform (IQ Enterprise®, BAXTER Laboratories), which enables the systematic interoperability of drug library data for patients treated at a tertiary University Hospital.

IQ Enterprise® is an essential tool for monitoring and continuously improving quality-of-care standards related to safe medication administration and has been available at the university hospital since late 2022. The sample was identified using this specialized software through non-probability convenience sampling, which included all infusion data administered via smart pumps for which information was available in the IQ Enterprise® across five adult ICUs: Medical ICU, Transplant ICU, Surgical ICU (Qx), Cardiovascular ICU (CV), and Coronary ICU (CC). A record of 35,738 medication infusions administered during the 12 months of 2023 was obtained, excluding other clinical care services. The sample was categorized into two main groups according to the drug library configured in the smart infusion pumps: the Medical/Transplant ICU group with 19,529 infusions and the Qx/CV/CC ICU group with 16,209 infusions.

For data analysis, a descriptive analysis was conducted using data extracted and exported from the IQ Enterprise® software, considering the number and type of infusions administered, and the percentages of compliance with drug libraries. Data were presented in tables using descriptive measures such as frequencies and proportions. Bar charts and scatter plots were used, depending on the data distribution, to display simple associations between key variables, such as medication type and compliance with dosing limits. Data were organized and analyzed using Microsoft Excel© and SPSS Statistics (Version 25). All collected data are freely available for access in Mendeley Data^16^.

**Ethical considerations:** This study was approved by the Research and Ethics Committee of the Fundación Cardioinfantil – LaCardio (Minutes No. 037, October 16, 2024).

## Results

The university hospital, Fundación Cardioinfantil–LaCardio, has five high-complexity adult ICUs and 67 beds available for the care of critically ill patients. Through the implementation and monitoring of the IQ Enterprise® software, data were exported that enabled the classification of the number of infusions administered across different adult ICUs, as well as the categorization of high-alert medications and antibiotics Table 1.

**Table 1:** Number of infusions per medication group

Medication groups	Intensive Care Units	Medications	Number of infusions
Vasoactive agents	Medical/Transplant ICU	Noradrenaline: 852 Vasopressin: 260 Adrenaline: 75	1,187
	Qx/CV/CC ICU	Noradrenaline: 1068 Vasopressin: 488 Adrenaline: 33	1,589
Inotropic agents	Medical/Transplant ICU	Dopamine: 7 Dobutamine: 66 Milrinone: 13 Levosimendan: 30	116
	Qx/CV/CC ICU	Dopamine: 1 Dobutamine: 343 Milrinone: 166 Levosimendan: 139	649
Sedatives and analgesics	Medical/Transplant ICU	Midazolam: 331 Propofol: 181 Dexmedetomidine: 389 Fentanyl: 438 Remifentanil: 100	1,439
	Qx/CV/CC ICU	Midazolam: 260 Propofol: 105 Dexmedetomidine: 436 Fentanyl: 442 Remifentanil: 5	1,248
Neuromuscular relaxants	Medical/Transplant ICU	Cisatracurium: 119	119
	Qx/CV/CC ICU	Cisatracurium: 37	37
Antibiotics	Medical/Transplant ICU	3,591 infusions	4,580
	Qx/CV/CC ICU	989 infusions	

Source: © 2024 Baxter. All rights reserved. Baxter and IQ Enterprise Connectivity Suite are registered trademarks of Baxter International Inc. Data exported by: [USER] on November 15, 2024, at 11:42:19 a.m. ICU: Intensive Care Unit, Qx/CV/CC ICU: Surgical, Cardiovascular, and Coronary Intensive Care Units.

 DERS compliance, referring to the drug library configured in the smart infusion pumps, was determined, as opposed to the basic mode, which consists of standard programming that does not allow identification of the medication being administered. During the first year of implementation and use of data interoperability, an overall compliance rate of 66% was observed across the institution's adult ICUs. The platform enabled visualization of overrides and hard-limit blocks, which refer to the number of times an operator performed or attempted to perform modifications to previously established parameters for different medications (soft and hard limits), with a total of 15,584 events identified. Overrides correspond to actions to modify medication doses outside standardized limits and do not affect the patient's health (soft limits), whereas hard-limit blocks are system-configured blocking actions (hard limits) that restrict dose adjustments for high-alert medications, thereby preventing patient harm Table 2.

**Table 2:** Drug library compliance

Service area	Number of infusions (n=35,738)	DERS % 66.06 (n=23,609)	Basic mode % 33.94 (n=12,129)	Overrides % 27.96 (n=9,995)	Hard-limit blocks % 15.63 (n=5,589)
Medical/Transplant ICU	19,529	67.21 (13,125)	32.79 (6,404)	61.14 (6,110)	44.93 (2,511)
Qx/CV/CC ICU	16,209	64.68 (10,484)	35.32 (5,725)	38.86 (3,885)	55.07 (3,078)

Source: © 2024 Baxter. All rights reserved. Baxter and IQ Enterprise Connectivity Suite are registered trademarks of Baxter International Inc. Data exported by: [USER] on November 15, 2024, at 11:42:19 a.m. DERS: Dose error-reduction system, ICU: Intensive Care Unit, Qx/CV/CC ICU: Surgical, Cardiovascular, and Coronary Intensive Care Units.

High-alert medications were classified as vasoactive agents, inotropic agents, sedatives and analgesics, and neuromuscular relaxants Table 3. Among these, a notable percentage of hard-limit blocks was observed by midazolam (40.52%), fentanyl (20.73%), and noradrenaline (14.88%). 

**Table 3:** Overrides and hard-limit blocks by medication (n=4,905)

Medication groups	Medications	Overrides % 63.02 (n=3,091)	Hard-limit blocks % 36.98 (n=1,814)
Vasoactive agents	Noradrenaline Vasopressin Adrenaline	20.96 (648) 11.91 (368) 1.49 (46)	14.88 (270) 3.91 (71) 1.21 (22)
Inotropic agents	Dopamine Dobutamine Milrinone Levosimendan	0.91 (28) 17.99 (556) 2.33 (72) 0.03 (1)	0.44 (8) 2.04 (37) 1.38 (25) 0.61 (11)
Sedatives and analgesics	Midazolam Propofol Dexmedetomidine Fentanyl Remifentanil	0.55 (17) 11.74 (363) 9.64 (298) 19.86 (614) 1.33 (41)	40.52 (735) 4.69 (85) 8.16 (148) 20.73 (376) 0.77 (14)
Neuromuscular relaxants	Cisatracurium	1.26 (39)	0.66 (12)

Source: © 2024 Baxter. All rights reserved. Baxter and IQ Enterprise Connectivity Suite are registered trademarks of Baxter International Inc. Data exported by: [USER] on November 15, 2024, at 11:42:19 a.m.

With regard to hard-limit blocks, the configuration of smart infusion pumps enabled the prevention of dosing errors involving high-alert medications, including those under special control. Considering attempts to exceed upper hard limits, it was identified that, for midazolam, the maximum configured dose was 15 mg/h, while the highest attempted dose resulting in a hard-limit block was 200 mg/h. For fentanyl, the established threshold was set at up to 500 mcg/h, with programming attempts ranging from 550 to 1200 mcg/h. For vasopressors, such as noradrenaline, the maximum permitted dose was 0.8 mcg/kg/min, with programming attempts ranging from 0.9 to 280 mcg/kg/min observed in the programming records. 

**Figure 1:**
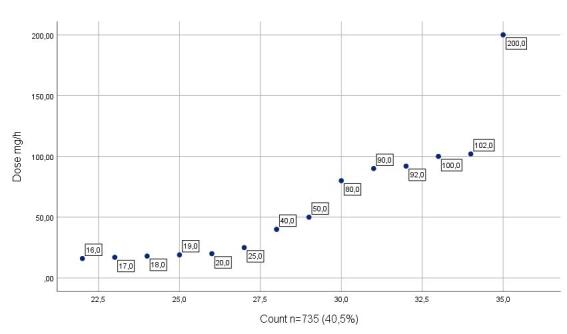
Midazolam hard-limit blocks

Finally, Figure 1, 2 , and 3  show the most relevant hard-limit blocks in high-alert medications identified through the monitoring platform. 

**Figure 2:**
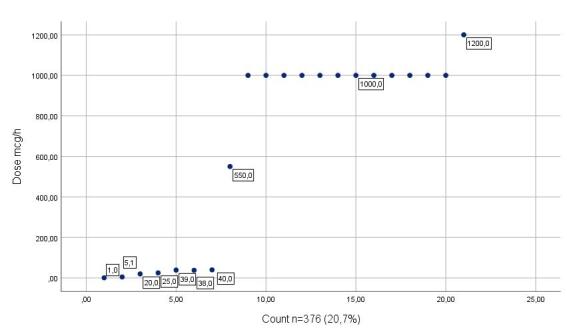
Fentanyl hard-limit blocks

**Figure 3:**
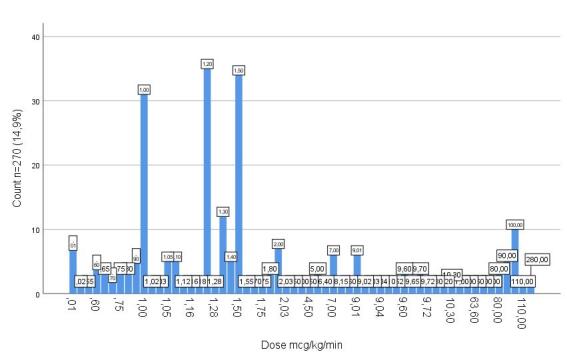
Noradrenaline hard-limit blocks

## Discussion

This study has demonstrated that the use of smart infusion pumps integrated with interoperable data- monitoring and analysis systems positively impacts patient safety. The results were consistent with existing literature at global and national levels, demonstrating drug library compliance ranging from approximately 65% to 80% during the first year of implementation; however, compliance may vary^15^,^17^.

The safe administration of medications in highly complex settings such as ICUs poses a challenge for professional nursing practice. For this reason, the implementation and adoption of technological tools that help minimize errors play a relevant role in clinical care. Previous studies have shown that ICUs are settings characterized by specialized care for complex patients, thereby generating significant demand for care, particularly the administration of multiple intravenous medications, including high-alert medications such as vasopressors, inotropic agents, sedatives, among other agents^15^,^18^.

Similar studies conducted in ICUs have reported the most relevant alerts, with vasoactive and sedative medications being of greatest concern. The findings confirm that high-alert medications such as noradrenaline, as well as certain specially controlled medications, such as midazolam and fentanyl, warrant particular attention, especially during dose titration. Through the integration of smart infusion pumps into a Dose Error Reduction System using the IQ Enterprise® software, a 76% prevention in potential adverse events related to doses exceeding those reported in the literature and in institutional protocols was demonstrated^19^.

Globally, numerous studies have linked technology to the prevention of medication administration errors; however, in Colombia, few studies have focused on analyzing this phenomenon^13^. Despite consistent in terms of compliance and potential errors prevented through technology, the results of this study raise concerns regarding a fundamental competency of nursing professionals, namely the safe administration of medications. Previous data suggest an incidence of errors in intravenous medication administration ranging from 49% to 81%, with ICUs representing one of the areas with the greatest contribution due to the high number of infusions administered, as well as the relationship between clinical complexity and high-alert medications^20^.

There is currently sufficient evidence to support the use of technologies related to medication administration for the benefit of patient safety; however, the process of implementing error reduction programs is not straightforward. Strategic planning, investment of time and money, standardization of medication and dosing practices by multidisciplinary teams (including pharmacists, specialized nurses, and physicians), development of drug libraries, training in the appropriate use of technology and library programming, adoption of the technology by nursing professionals, and the promotion of a safety culture related to medication administration are required. In addition, ongoing monitoring is necessary to allow for adjustments or the incorporation of new medications. Together, these requirements pose a challenge for institutions and health systems in terms of quality of care^21^–^25^.

Limitations: those related to sample selection and potential failures in data reception arising from interoperability between smart infusion pumps and the monitoring platform (IQ Enterprise®). For the purposes of the study, only data from the first year of the drug library implementation were analyzed, focusing on compliance and errors related to high-alert medications in terms of dose programming

## Conclusions

This study found a 66% compliance rate with the drug libraries integrated into smart infusion pumps across five adult ICUs during the first year of use. The configuration of hard limits contributed to reducing clinical practice errors involving high-alert medications such as midazolam, fentanyl, and noradrenaline.

The findings support the conclusion that the implementation of institutional monitoring programs for the safe medication administration, supported by technologies such as DERS integrated into smart infusion pumps, contributes to reducing errors in clinical practice, with a positive impact on patient safety, institutional continuous improvement processes, and the strengthening of the professional role of nursing.
